# Stable isotope measurements show increases in corn water use efficiency under deficit irrigation

**DOI:** 10.1038/s41598-018-32368-4

**Published:** 2018-09-20

**Authors:** Youjie Wu, Taisheng Du, Yusen Yuan, Manoj K. Shukla

**Affiliations:** 1 College of Engineering, Hunan Agricultural University, Changsha, 410128 China; 2 Center for Agricultural Water Research in China, China Agricultural University, Beijing, 100083 China; 3 Plant and Environmental Sciences Department, New Mexico State University, Las Cruces, New Mexico 88003 USA

## Abstract

Deficit irrigation has usually improved crop water use efficiency (WUE), but there are still gaps in our understanding of the mechanisms. Four irrigation treatments were a conventional furrow irrigation (CFI), border irrigation (BI), alternate furrow irrigation (AFI), and an AFI_(M/2)_ (the amount of irrigation was 50% of the AFI). The volume of irrigation water applied were nearly the same for CFI, BI, and AFI. The isotope (δ^18^O and δD) method was used to quantify corn root water uptake (RWU) during 2013–2014. Compared to CFI and BI, corn yield and WUE were 17.0-30.2% and 13.3-33.8% higher in AFI, respectively. No significant yield reduction were observed between AFI and AFI_(M/2)_. Corn RWU was more from deeper soil with increasing growth stage for AFI_(M/2)_, AFI, and CFI, but from shallower depth for BI. The depth for RWU varied in the order of AFI_(M/2)_  > AFI > CFI > BI. The maximum root density was in the depth of 40–80 cm at the growing stage in AFI, and 4–26% more water was extracted from the wetter and deeper root zones. The WUE increased under deficit irrigation, and stimulated the root growth with attendant decreases in water loss to deep percolation.

## Introduction

Deficit irrigation methods, such as alternate furrow irrigation (AFI), alternate partial root zone irrigation (APRI), and partial root zone drying (PRD), are recognized as agronomic practices that improve crop water use efficiency^1–5^. When compared with conventional surface irrigation methods, such as conventional furrow irrigation (CFI) and border irrigation (BI), AFI improves plant water use efficiency (WUE) without significant yield reductions^6^. Compared to conventional surface irrigation, APRI is reported to require 38.4% less irrigation water, with only slight decreases in shoot (5.9%) and total dry biomass (6.7%) but a 24.3% increase in WUE^7^. Other studies have reported that APRI can save 25–75% of irrigation water, depending on the crop growth stages, without significantly affecting crop yield^8^. APRI is also reported to improve WUE by 20–29% for corn and 61–66% for wheat^9^. Previous research of deficit irrigation were focused on crop yield, water savings, and plant physiology. But the mechanisms in improving water use efficiency under deficit irrigation not studied in great details.

High WUE in APRI is due to the reduction in stomatal opening and a better balance between reproductive growth and vegetative growth during plant development^2,3,10–13^. However, determination of the causes of enhanced crop yields and WUE under partial root zone drying remains challenging^14^. Few studies have reported the mechanisms involved in improving WUE by analyzing plant root water uptake (RWU) patterns. RWU based on the proportion of water absorbed from each soil layer and each root zone and their role in improving the WUE under different irrigation regimes have not been fully investigated.

Plant RWU depends on the available soil water, root density, and root distribution^15–19^. Some studies have indicated that due to the large spatial variations in the root distributions and soil moisture, these two alone do not necessarily explain the extraction depth and volume of soil water obtained by plants^20^. Traditional methods, such as digging roots for studying RWU, have many practical difficulties, provide little insight into water uptake patterns, and may be unreliable indicator of water use in the space-time scale^21^. Stable isotope techniques are effective for understanding plant RWU patterns^22–25^.

Stable oxygen (δ^18^O) and hydrogen (δD) isotopes have been widely used to determine plant RWU^23,26–28^. RWU of plant is a mixture of water from different root zones, and it can be assessed by using isotopic compositions of water in plants as well as in different soil layers^29,30^ because there is no stable isotope fractionation during the absorption of soil water by plant roots^28,29^. The naturally occurring vertical gradients of stable isotopes in the soil profile provide important information on RWU from soil layers^26,31^. Using a stable isotope technique, it was reported that soil water from shallow soil depths was taken up by plants mainly during the wet season, while soil water uptake from deeper soil depths occurred mainly during the dry season^20^. Corn obtained 45% of water mainly from 0–20 cm soil layer^32^, and with increasing plant growth isotope analysis showed increases in water extraction depths^27,30^.

In this study, we used a stable isotope technique under four irrigation regimes (AFI, BI, CFI, and AFI_(M/2)_ [the amount of irrigation was 50% of the AFI]). Combined with corn root distribution and soil moisture, we assessed the RWU patterns using the IsoSource model. The objectives of this study were to: (1) quantify corn RWU from each soil layer and root zone at various growth stages under each irrigation regimes, and (2) identify mechanisms of WUE improvement under deficit irrigation. It can improve our understanding of corn RWU processes and the mechanisms related to WUE.

## Materials and Methods

### Study site

The study was conducted during 2013–2014 at Shiyanghe Experimental Station of China Agricultural University, Wuwei City, Gansu Province, China (37°52′ N, 102°51′ E). The experimental area has a typical continental temperate climate, mean annual sunshine duration 3000 h, and long-term average temperature of 8 °C. The mean annual precipitation for the region is 164 mm, mean annual evaporation is 2000 mm (from free water surface), and groundwater table varies between 30 and 40 m from soil surface. Soil texture varies from loam to sandy loam, with average field capacity water contents of 0.28 and 0.26 cm^3^/cm^3^ ^33^, respectively.

### Experimental design

**Figure 1:**
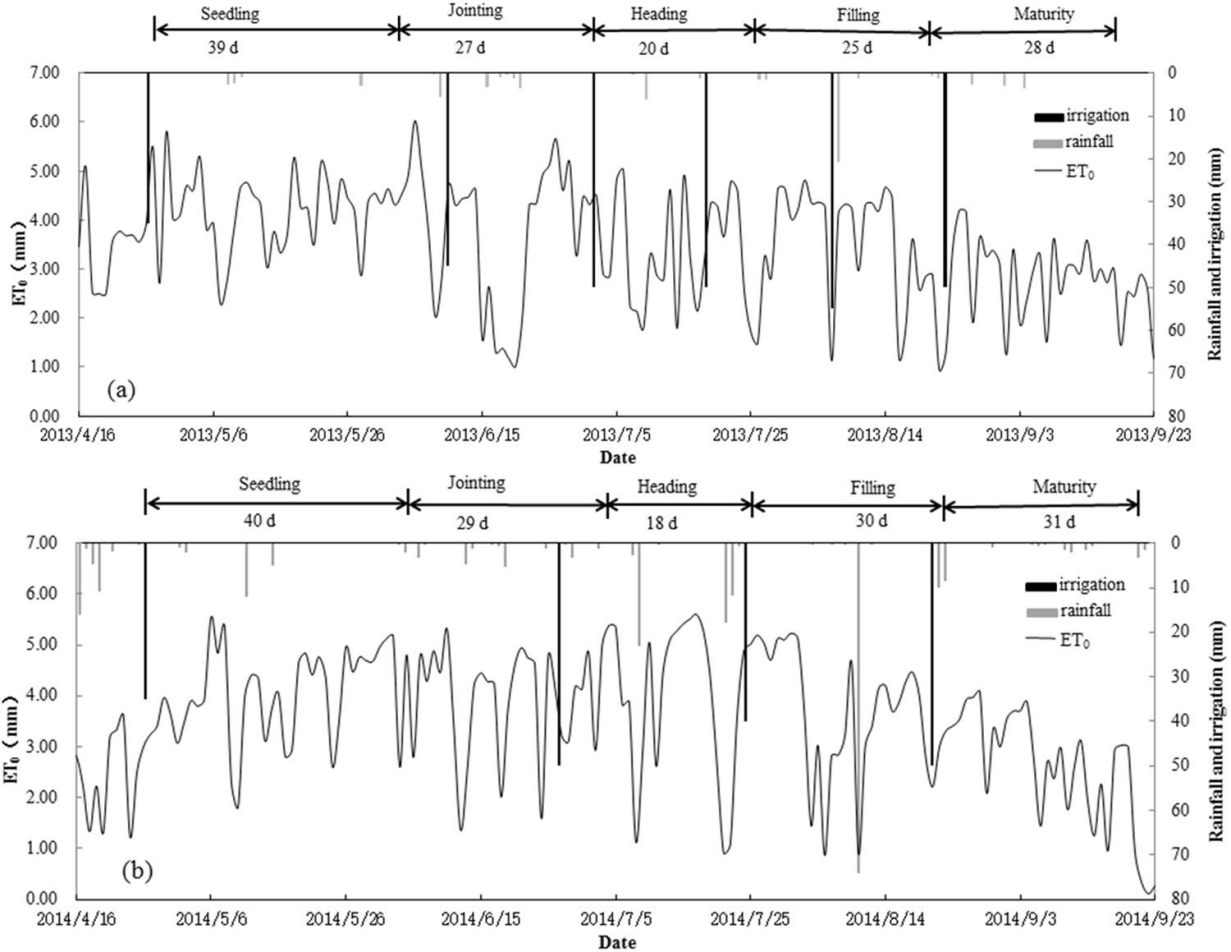
The rainfall, irrigation quota, and ET_0_ during all growing stages of corn. (**a**) 2013, (**b**) 2014.

Three surface irrigation treatments applied were AFI (alternate furrow wetted during irrigations), CFI (all furrows wetted with every irrigation), and BI (similar to flood irrigation). Total irrigation volumes applied to the three irrigation treatments were the same, and an additional alternate furrow irrigation treatment (AFI_(M/2)_) was designed where the applied irrigation was 50% of the AFI. Rainfall, irrigation, and ET_0_ during all growing stages of corn are presented in Fig. 1. The irrigation schedule was done following the method described in^34^.

**Figure 2:**
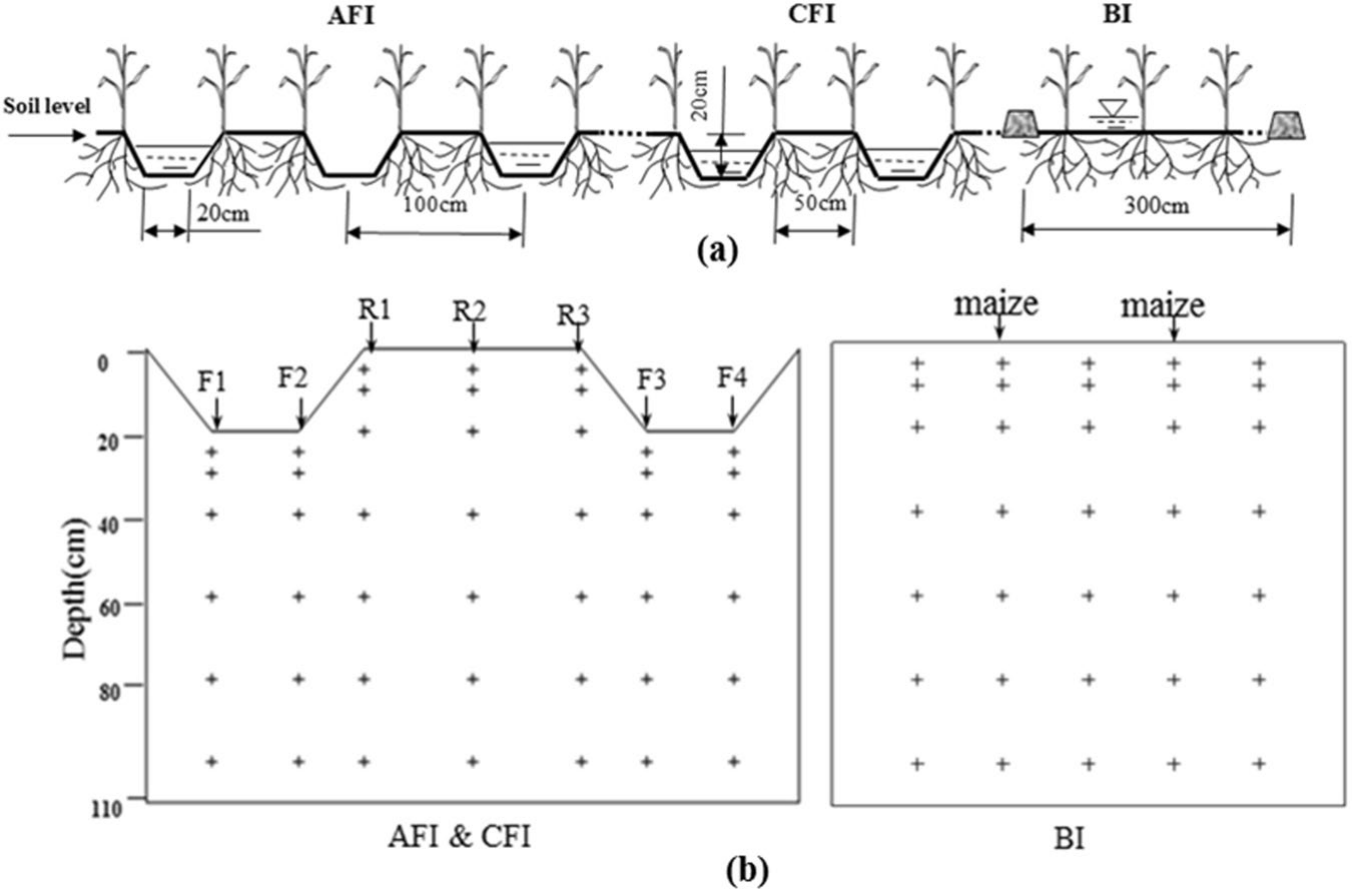
(**a**) Sketch of the three different irrigation methods (AFI, CFI, and BI). (**b**) Soil sampling sites in the two-dimensional profile. From south to north were labeled as F1, F2, R1, R2, R3, F3, and F4. R, ridge; F, furrow; +, soil samplings from each layer and zone.

For the irrigation treatments of AFI, CFI, and AFI_(M/2)_, corn was planted on both sides of the ridge (Fig. 2). For the BI, corn planting density and management were similar to other treatments with a density of 66,000 plants per hectare. The field experiment consisted of 12 plots with three replications per treatment. Each plot was 4 m × 60 m for the furrow irrigations and 3 m × 60 m for the border irrigation in 2013. However, the plot size was changed to 4 m × 100 m and 3 m × 100 m for furrow and border irrigations in 2014, respectively.

### Sampling and measurements

Soils samples were obtained using an auger from the depths of 0–5, 5–10, 10–20, 20–40, 40–60, 60–80, and 80–110 cm at different growth stages of corn. The stems were also collected on the day of the soil sampling between 12:00 and 14:00 hours on sunny days to avoid the isotope fractionation. Water was extracted from soil and plant samples using a vacuum extraction system (LI-2000, LICA, China). Irrigation water and precipitation samples were collected, filtered^25^, and stored in airtight containers at 4 °C. Samples were analyzed for water-stable isotopes (δD and δ^18^O) by Water Isotope Analyzer (PICARRO L2130i, Picarro, USA). The isotopic composition relative to the standard mean ocean water (SMOW) was calculated as follows:

1 $${\rm{\delta }}D=(\frac{{(D/H)}_{sample}}{{(D/H)}_{standard}}-1)\times 1000$$

2 $${{\rm{\delta }}}^{18}{\rm{O}}=(\frac{{({18}_{O}/{16}_{O})}_{sample}}{{({18}_{O}/{16}_{O})}_{standard}}-1)\times 1000$$

where (D/H)_sample_, (^18^O/^16^O)_sample_, (D/H)_standard_, and (^18^O/^16^O)_standard_ are the molar ratios of hydrogen and oxygen in the sample and standard water, respectively. The analytical precision was <0.1‰ for δ^18^O and <2.0‰ for δD.

Soil moisture content determined gravimetrically was multiplied by the soil bulk density to obtain volumetric soil moisture content. The soil and water samples were collected at the same time and from nearby locations for isotope analysis.

Crop evapotranspiration (ET; mm) was calculated using the water balance equation:

3 $${\rm{ET}}={\rm{P}}+{\rm{I}}+{W}_{0}+{W}_{h}$$

where *P* is the total rainfall during the growing period (mm), *I* is the amount of irrigation water (mm), and *W*_*0*_ and *W*_*h*_ are the amount of soil water (mm) stored at 1.1 m depth during planting and harvesting stages, respectively.

The total water use efficiency (WUE_ET_; kg ha^-1^mm^-1^) and irrigation water use efficiency (WUE_I_; kg ha^-1^mm^-1^) were estimated as:

4 $$WU{E}_{ET}=\frac{GY}{ET}$$

5 $$WU{E}_{I}=\frac{GY}{I}$$

where *ET* is total water used (mm), *I* is irrigation water amount (mm), and *GY* is the grain yield (kg ha^-1^) harvested for all the treatments and plots; final results were estimated by the total grain yield.

Four corn plants per treatment were selected, and root samples were collected with a root auger (diameter 8 cm) to a depth of 100 cm in 10-cm increments. The root length density (RLD) (cm/cm^3^) was calculated by dividing the total root length (cm) with the volume (cm^3^) of the soil, separately by depth.

### Statistical analyses

RWU of corn from each root zone was estimated by IsoSource method^35^; the method was based on multi-source mass balance assessment. The soil profile (0–110 cm) was divided into five parts: 0–20, 20–40, 40–60, 60–80, and 80–110 cm. The mass balance equation was:

6 $${\rm{\delta }}{D}_{P}=\sum _{i=1}^{5}{f}_{i}\times \delta {D}_{i}$$

7 $${{\rm{\delta }}}^{18}{O}_{P}=\sum _{i=1}^{5}{f}_{i}\times {{\rm{\delta }}}^{18}{O}_{i}$$

8 $$1=\sum _{i=1}^{5}{f}_{i}$$

where f_i_ is the proportion of source *i* to the system; δD_p_ and δ^18^O_p_ are the plant stem hydrogen and oxygen isotope compositions, respectively; and δD_i_ and δ^18^O_i_ are the *i*^th^ soil layer hydrogen and oxygen isotope compositions, respectively. The range of potential contributions of water from each soil layer to corn RWU can be estimated by the multiple-source mass balance assessment. The results are reported as the distribution of feasible solutions (i.e., minimum–maximum)^35^.

The potential contribution of water source (water in different soil layers and zones) was increased incrementally, and the isotopic mass-balance was performed at each increment^32^. The number of feasible combinations (N) was calculated as:

9 $${\rm{N}}=\frac{[(100/j)+(S-1)]!}{(100/j)!(S-1)!}$$

where *j* is increment (%) and *S* is the number of sources. In this study, the increment and tolerances were set as 1% and 0.01‰, respectively.

## Results

### Yield and water use efficiency of corn

Corn yield under AFI was significantly higher than other treatments for both years (Table 1). Compared to BI, no significant yield reductions were observed between BI and AFI_(M/2)_, even though only half of the irrigation water was applied to AFI_(M/2)_ compared to BI during both years. A comparison with CFI showed that 17.0% and 30.2% more corn yield was obtained with AFI during 2013 and 2014, respectively. Compared to BI, 24.5% and 30.4% more corn yield was obtained with AFI during 2013 and 2014, respectively. The highest WUE_ET_ was obtained for AFI_(M/2)_ during 2013 and for AFI during 2014. Meanwhile, the WUE_I_ was highest in AFI_(M/2)_ during both years. In contrast, the lowest WUE_ET_ and WUE_I_ were obtained for BI during both years. AFI produced the highest yield and improved WUE by 13.3–33.8%, while no significant differences in WUE_ET_ and WUE_I_ were observed among CFI and BI.

**Table 1:** Yield and WUE of corn under different irrigation regimes.

Year	Treatments	Yield (kg ha^−1^)	Rainfall (mm)	ET (mm)	WUE_ET_ (kg ha^−1^mm^−1^)	WUE_I_ (kg ha^−1^mm^−1^)
2013	AFI	6139.79 a	68.4	374.5 a	16.39 b	21.54 b
	CFI	5247.53 b	68.4	362.6 a	14.47 bc	18.41 c
	BI	4931.82 bc	68.4	363.6 a	13.56 c	17.30 c
	AFI_(M/2)_	4201.62 c	68.4	238.2 b	17.64 a	29.38 a
2014	AFI	7397.08 a	241.0	476.9 b	15.51 a	42.27 b
	CFI	5681.23 b	241.0	489.4 ab	11.59 c	32.46 c
	BI	5673.03 b	241.0	493.7 a	11.51 c	32.42 c
	AFI_(M/2)_	5105.02 b	241.0	389.5 c	13.01 b	57.97 a

Letters following the numbers indicate statistical significance within the same column at P_0.05_ level.

WUE_ET_ is total water use efficiency and WUE_I_ is irrigation water use efficiency. AFI, CFI, BI, and AFI_(M/2)_ represent alternate furrow irrigation, conventional furrow irrigation, border irrigation, and alternate furrow irrigation with half of the irrigation water, respectively.

### Stable hydrogen and oxygen isotopic composition (δD and δ^18^O)

Mean values of δ^18^O and δD for precipitation were −4.05‰ and −19.42‰ in 2013 and 2014, respectively (Table 2). The slope and intercepts for the best fit linear regression between δD and δ^18^O for the local meteoric water line (LMWL) in 2013 and 2014 (δD = 7.2 δ^18^O + 9.7) were close to those for the global meteoric water line (GMWL; δD = 8 δ^18^O + 10)^36^ with an R^2^ of 0.93. The slope and intercept values for LMWL are less than those for the GMWL. The slope and intercept values for soil water line (δD = 6.0 δ^18^O–6.9) were also less than those for the GMWL, reflecting a strong evaporation effect on soil water.

**Table 2:** Isotope compositions of water samples and relationship between δD and δ^18^O during 2013 and 2014.

Samples		δ^18^O (‰)			δD (‰)			Linear relation
		Min	Max	Mean	Min	Max	Mean	
Precipitation		−9.71	3.34	−4.05	−63.59	22.05	−19.42	δD = 7.2 δ^18^O + 9.7 (R^2^ = 0.93, n = 44)
Soil Water	0–5 cm	−12.43	3.23	−3.24	−81.97	17.66	−20.36	δD = 6.0 δ^18^O −6.9 (R^2^ = 0.94, n = 557)
	5–10 cm	−11.60	−0.87	−5.45	−75.04	−8.49	−34.71	
	10–20 cm	−12.38	−1.33	−6.82	−85.29	−13.10	−44.58	
	20–40 cm	−11.50	−3.14	−8.58	−75.60	−24.61	−55.37	
	40–60 cm	−13.97	−4.53	−9.37	−87.85	−29.21	−60.81	
	60–80 cm	−13.27	−6.34	−9.78	−86.17	−34.77	−68.07	
	80–110 cm	−14.38	−7.71	−10.84	−94.17	−46.24	−76.76	
Stem water		−13.38	−7.80	−10.55	−93.01	−50.07	−71.87	δD = 5.9 δ^18^O − 7.8 (R^2^ = 0.93, n = 89)
Irrigation water		−13.41	−7.77	−10.04	−86.52	−52.42	−68.73	δD = 5.7 δ^18^O − 8.4 (R^2^ = 0.96, n = 26)

**Figure 3:**
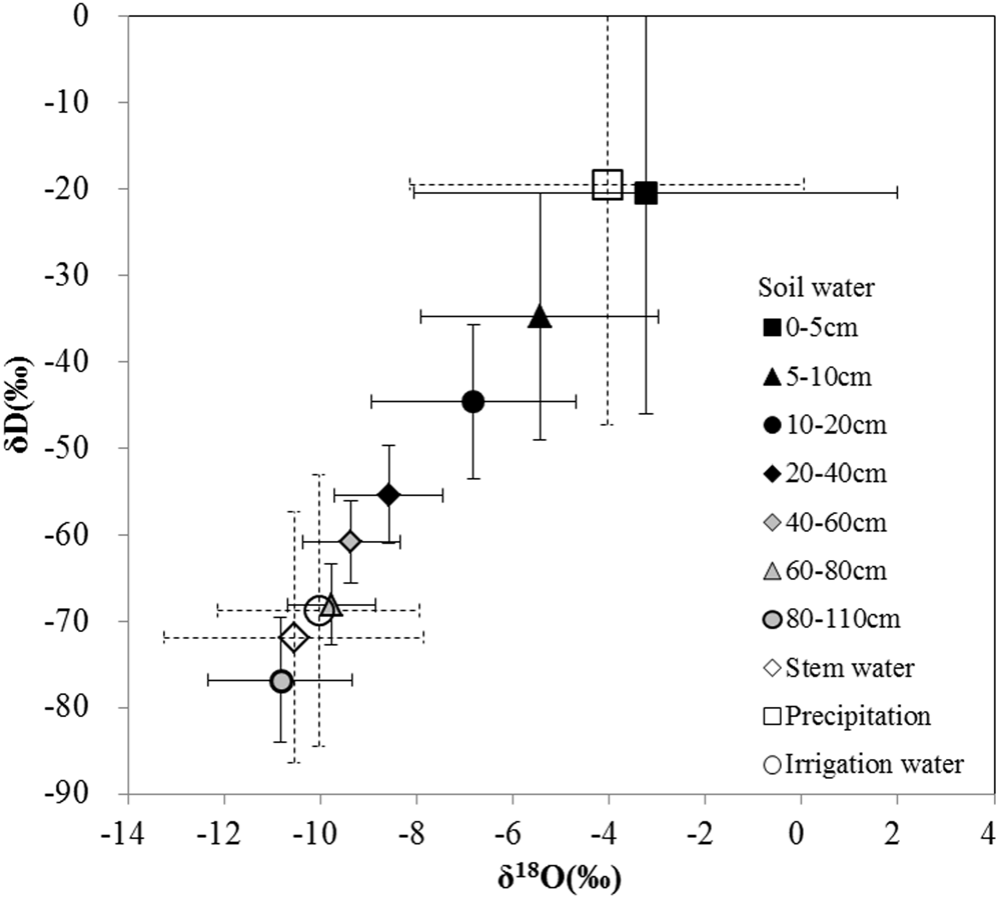
Relationship between δD and δ^18^O of water samples during 2013 and 2014.

Mean values of δ^18^O and δD for soil water at 0–20 cm depth were −4.98‰ and −36.06‰ in 2013 and 2014. Mean values of δ^18^O and δD for soil water at 20–110 cm depth were −8.89‰ and −59.19‰, respectively. The higher means and errors in the surface soil layer (Fig. 3) than deeper layers indicated isotopic fractionation due to evaporation of the shallow soil water^37,38^. The best-fit linear regression line between δD and δ^18^O for the corn stem water (δD = 5.9 δ^18^O–7.8) were close to the soil water line. Similar values obtained for δD and δ^18^O in stem water and irrigation water indicated that the potential water source for corn was from the soil and irrigation water (Fig. 3). The mean values ranged from −86.52‰ to −52.42‰ for δD and −13.41‰ to −7.77‰ for δ^18^O for both years (Table 2).

In this study, only δ^18^O data were further analyzed because the distribution of hydrogen and oxygen isotopes showed a good uniformity, and the fractionation of δ^18^O was less significant than that of δD.

### Corn water uptake pattern

**Figure 4:**
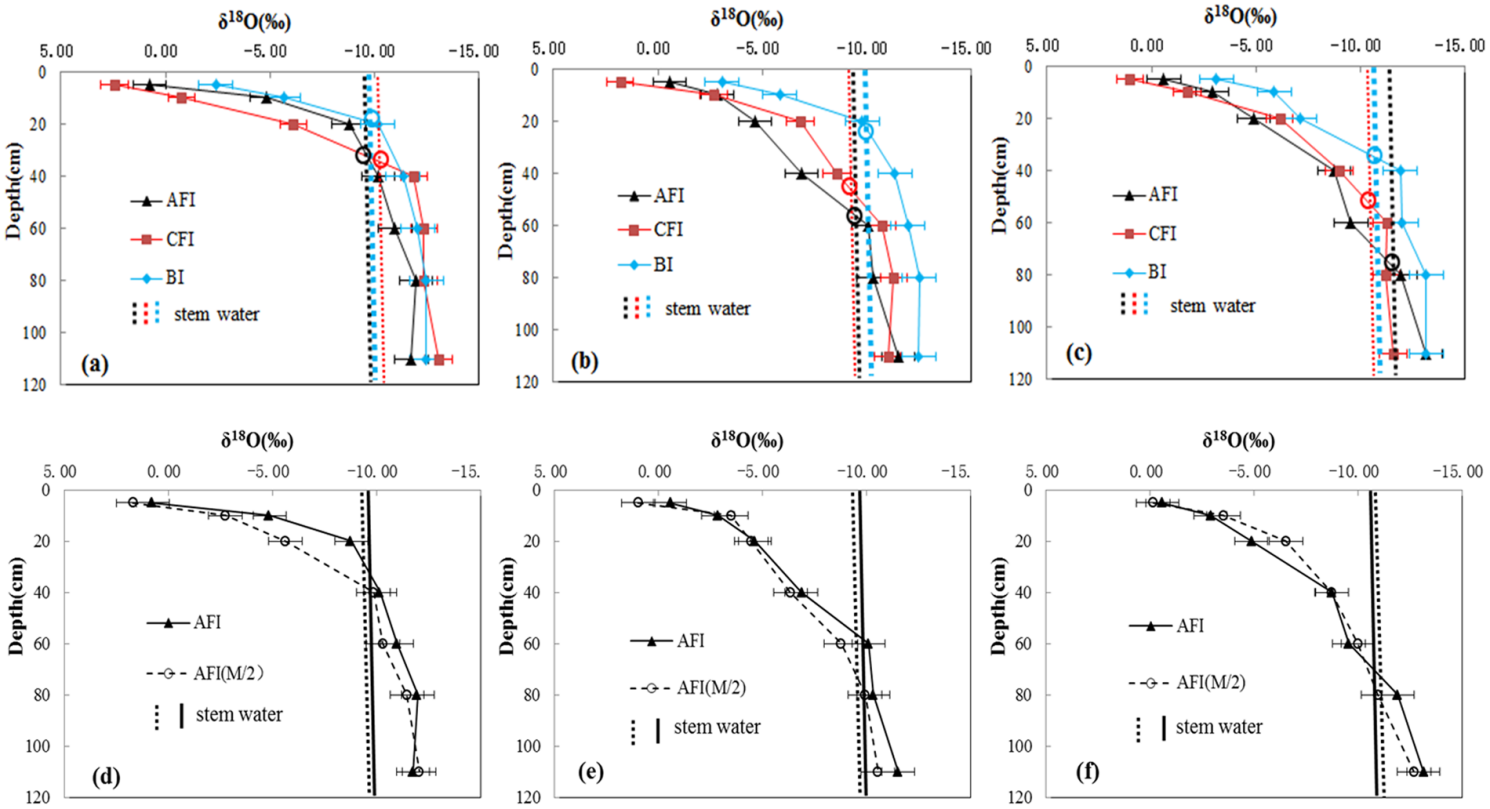
δ^18^O of soil water and corn stem water under different irrigation regimes. (**a,d**) Jointing, (**b,e**) heading, and (**c,f**) filling stage. (Based on the theory that if the soil water isotope composition of one certain layer is similar to the plant stem water, it can indicate that this layer of soil water was mainly absorbed by plant roots).

The δ^18^O in the soil water profile decreased with depth (Fig. 4). The depth, corresponding to the point of intersection between δ^18^O in the soil water profile and stem water line, was considered to be the depth from where water uptake occurred (Fig. 4a–c). At this depth, there was no stable isotope fractionation during plant water uptake, and the isotopic composition of water was consistent with that of the stem water^23^. At the jointing stage, the intersection between δ^18^O in stem water and soil water for BI treatment was between 0 and 20 cm soil depth and for AFI and CFI, it was between 20 and 40 cm depth (Fig. 4a). Thus, corn RWU was mainly from the 0–20 cm layer under BI and from 20–40 cm layer under AFI and CFI at the jointing stage. At the heading stage, the intersection from 0–20 and 40–60 cm depths for three irrigation regimes (Fig. 4b) suggested that corn mainly used water from the 0–20 cm depth under BI and from 40–60 cm depth under AFI and CFI. At the filling stage, corn mainly absorbed water from the 20–40, 40–60, and 60–80 cm depths under BI, CFI, and AFI, respectively (Fig. 4c).

Depth for corn RWU for AFI_(M/2)_ increased with the growth stage. At each growth stage, corn RWU for AFI_(M/2)_ was from deeper soil depths than AFI. For example, corn RWU for AFI_(M/2)_ was from 60–80 and 80–100 cm depths at the heading and filling stage, respectively, while for AFI it was from 40–60 and 60–80 cm depths (Fig. 4d–f).

**Figure 5:**
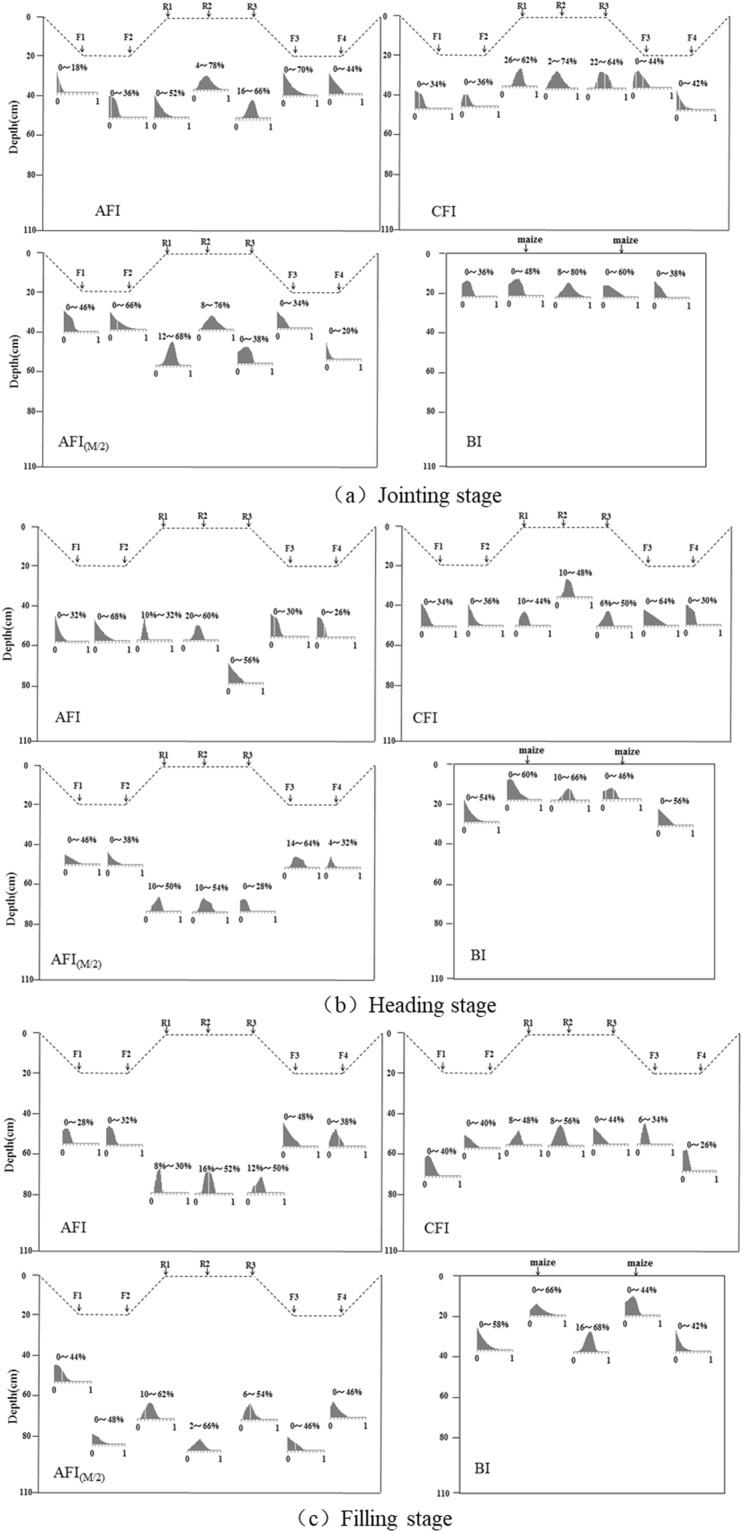
Frequency histograms produced from the IsoSource method (multi-source mass balance) showing estimated proportion of total corn water uptake from each root zone of two-dimensional soil profile under different irrigation regimes. (**a**) Jointing stage, (**b**) heading stage, and (**c**) filling stage. (Results are average for the growing stage of corn).

In the frequency histograms produced from the IsoSource method (multi-source mass balance, results are average for the growing stage of corn), corn mainly used water from the soil zones where the histogram patterns were relatively convergent meaning they had a crest value (Fig. 5). For example, histogram patterns were relatively convergent at the 20–40 cm soil depth and below the ridge (R2 and R3 as shown in Fig. 5a, AFI), indicating that corn primarily used water from the 20–40 cm depth at the jointing stage. For AFI, about 4–78% and 16–66% of corn RWU was accounted for from the ridge (R2, R3) with the crest values of 38% and 48%, respectively. Thus, approximately 38% and 48% of water was mainly absorbed from the 20–40 cm depth below R2 and R3 under AFI. With 2–74% and 22–64% of RWU from the 20–40 cm depth below R2 and R3, CFI had the highest proportion RWU of 34% and 42%, respectively (Fig. 5a, CFI). Similarly, approximately 44% and 36% of water was absorbed from the 20–60 cm depth below R1 and R2 under AFI_(M/2)_ (Fig. 5a, AFI_(M/2)_), and 40% from the 0–20 cm depth under BI (Fig. 5a, BI).

At the heading stage for AFI (Fig. 5b), the RWU of corn was primarily from the 40–60 cm depth, accounting for 22% and 40% from F2 and R2, respectively (Fig. 5b, AFI); and 30% and 34% from R2 and R3 at the 20–60 cm depth, respectively, for CFI (Fig. 5b, CFI). For AFI_(M/2)_, RWU was 32% from both R2 and F3 at the 40–80 cm depth(Fig. 5b, AFI_(M/2)_); and for BI, it was 46% from the 0–20 cm depth (Fig. 5b, BI).

At the filling stage, the RWU for AFI was primarily from the 40–80 cm depth, accounting for 36% and 38% from R2 and R3, respectively (Fig. 5c, AFI). It was 34% and 36% from R1 and R2 at the 40–60 cm depth, respectively for CFI (Fig. 5c, CFI); 36% and 40% from R1 and R2 at the 60–100 cm depth, respectively for AFI_(M/2)_ (Fig. 5c, AFI_(M/2)_); and 50% from the 0–40 cm depth for BI (Fig. 5c, BI).

### Root distribution and soil moisture profile

**Figure 6:**
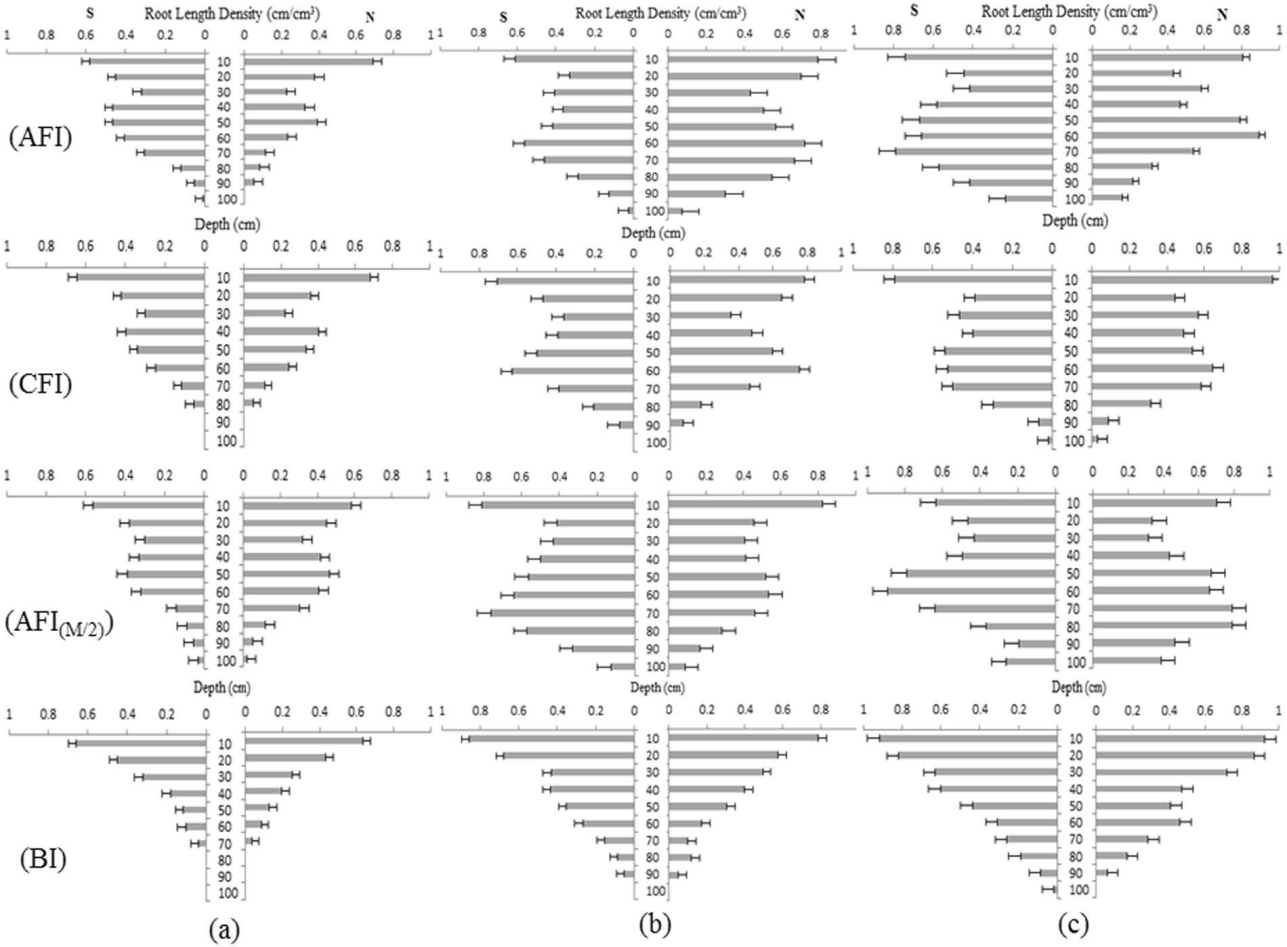
Corn root distribution under the four irrigation regimes at different growth stages. (**a**) Jointing stage, (**b**) heading stage, and (**c**) filling stage. S, south of the center of the ridge (or corn for BI); N, north of the center of the ridge (or corn for BI).

Spatial distribution of the soil profile was influenced by trenching and furrows construction for AFI, CFI, and AFI_(M/2)_, and those also influenced the soil profile wetting and root distribution within 0–10 and 40–80 cm depths (Fig. 6). At the heading stage, the maximum root length density (RLD) was obtained at 0–10 and 60–70 cm depths for AFI, 0–10 and 50–60 cm depths for CFI, and 0–10 and 60–70 cm depths for AFI_(M/2)_. However, maximum RLD occurred only at the 0–20 cm depth for BI (Fig. 6b). The maximum depth of roots for AFI_(M/2)_ was greater than that for AFI, CFI, and BI during each growth stage. For example, at the jointing stage, roots were found up to 100 cm deep for AFI_(M/2)_, 90 cm for AFI, 80 cm for CFI, and 70 cm for BI (Fig. 6a). Compared with CFI, corn root distribution south and north of the ridge center was asymmetric under AFI and AFI_(M/2)_, but it was symmetric under BI. AFI changed the soil profile wetting and stimulated deeper and wider root growth in the dry side of the profile^3,39^.

**Figure 7:**
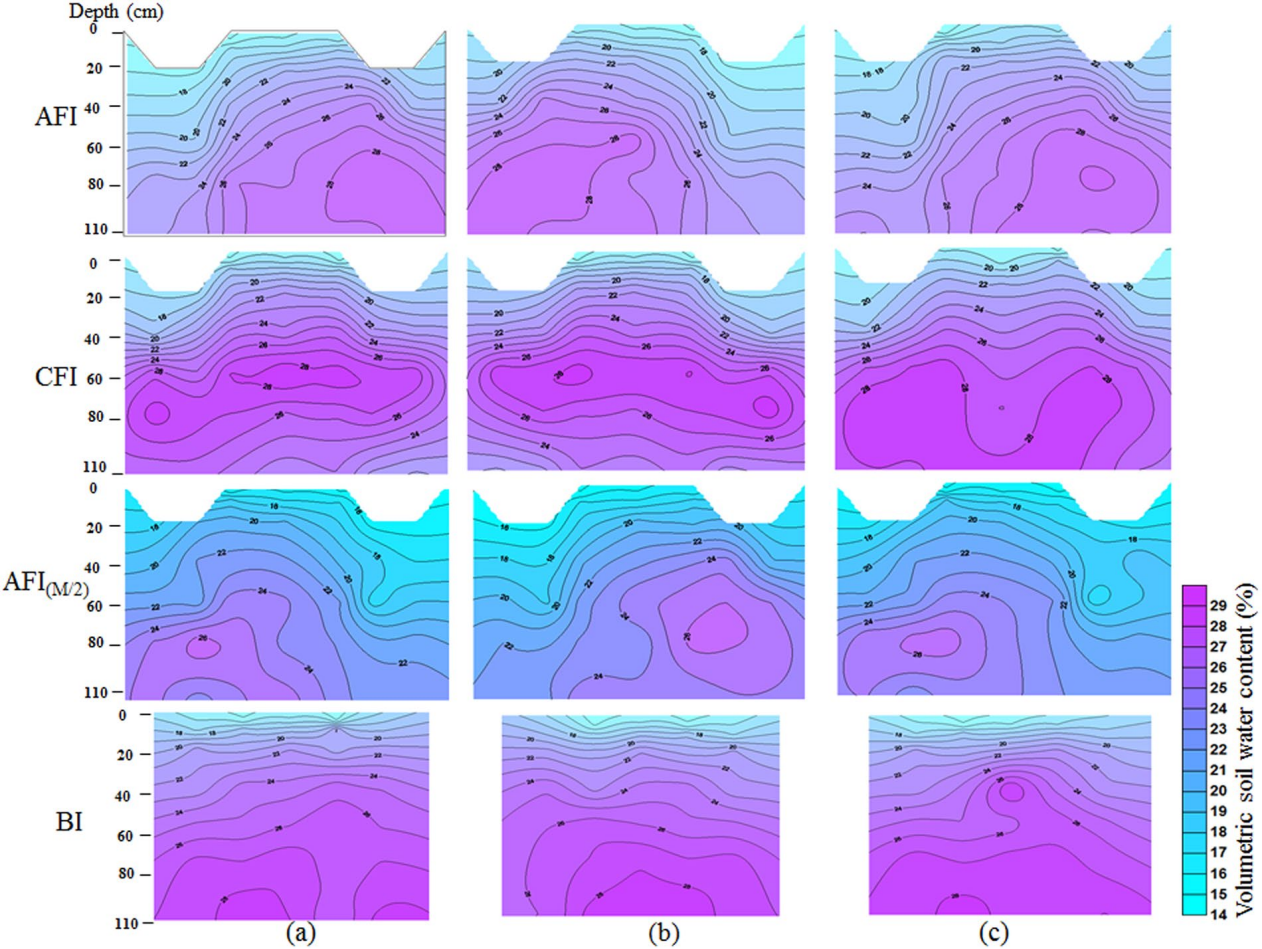
Soil moisture profile under the four irrigation regimes at different growth stages. (**a**) Jointing stage, (**b**) heading stage, and (**c**) filling stage. The sampling time for soil moisture was consistent with the samples of root and soil water isotopes.

The soil moisture profiles for the three corresponding periods under each of the four irrigation regimes are shown in Fig. 7. For AFI, the soil water distribution in two-dimensional profile was not symmetric. The two-dimensional distribution of the soil water profile in AFI_(M/2)_ was similar to AFI, but water content was less than AFI at most depths. For CFI, the soil moisture was greatest at the 60–90 cm depth, it increased asymptotically for BI with increasing soil depth, and was near field capacity water content (28%) below 80 cm.

### Corn water uptake pattern of two-dimensional soil profile within a short irrigation cycle

**Figure 8:**
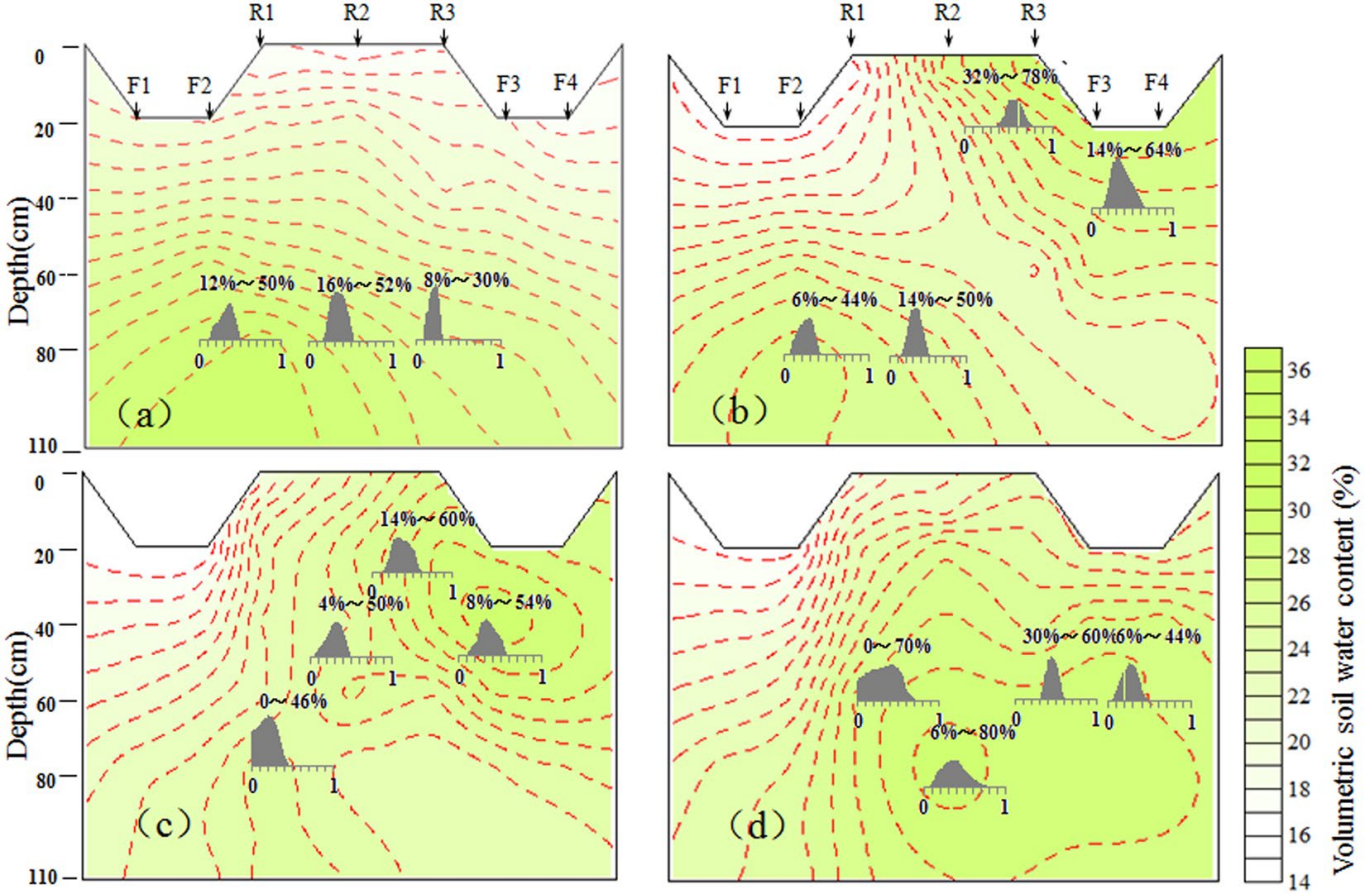
Soil moisture profile and frequency histograms of corn water uptake from different soil zone under AFI. (**a**) 1 d before irrigation, (**b**) 1 d after irrigation, (**c**) 3 d after irrigation, and (**d**) 7 d after irrigation.

Soil water content was higher at deeper soil depths one day prior to the irrigation (Fig. 8a), and corn RWU was 12–50% from the 60–80 cm depth of R1 (36%) and 16–52% from the 60–80 cm depth of R2 (38%) (Fig. 8a). One day after the irrigation, with a higher soil moisture in the top soil depths of the irrigated furrows, 32–78% and 14–64% of water were extracted from the 0–20 cm depth of R3 and 20–40 cm depth of F1, respectively (Fig. 8b). Three days after the irrigation, about 32% and 34% of water was taken up from the 20–40 cm depth of R3 and 40–60 cm depth of F1, respectively (Fig. 8c). Seven days after the irrigation, with 0–70% and 6–80% of water uptake were from the 40–60 cm depth of R1 and 60–80 cm depth of R2, respectively (Fig. 8d). Therefore, combining the soil moisture profile with RWU patterns within the irrigation cycle showed that corn RWU depths varied with the change in soil moisture. In AFI, more water was taken up from the wetter (irrigated) part of the root zones, which can save water by reduced deep percolation loss.

## Discussion

### Stable isotope techniques for quantifying crop water uptake

In our study, the slope and intercept values in the regression equation for soil water were smaller than those for the local meteoric water line (LMWL), indicating a strong kenetic fractionation and a weak equilibrium fractionation respectively, infering a strong evaporation effect on soil water during water uptake by crops. Comparison between the isotopic composition of soil water and stem water indicated that corn stem water was taken up from soil water from different depths under different irrigation treatments (Fig. 4). Results of the direct inference approach were consistent with the multi-source mass balance assessment (Fig. 5) as well as with published references^18,30,40^. The stable isotope analyses improved our understanding of corn RWU processes and active rooting zones^21^. Using stable isotope analyses, Asbjornsen *et al*.^32^. confirmed that corn obtained up to 45% of water from shallow soil depths (0–20 cm), which was similar to our result under BI. Therefore, stable isotope techniques provided an effective quantifying method for explaining soil and plant water dynamics. Traditional methods, such as chemistry solutions of the soil^41^, analysis of a plant’s root system^20^, and sap flow^42,43^, can not distinguish these effects. Meanwhile, the sources of error in this isotope-based study may come from the variability of isotopic signatures, sample size and time, and weather conditions. It is still a great challenge to accurately calculate RWU. We expect that future refinement of methods would produce accurate assessment of soil and plant water dynamics.

### Root distribution and soil moisture profile under different irrigation regimes

In this study, the water uptake by the corn roots occurred from deeper soil layer with the increasing growth stages in AFI, AFI_(M/2)_, and CFI, but it was from shallow soil depths under BI. At the same time, depth of corn RWU under four irrigation methods varied in the order AFI_(M/2)_ > AFI > CFI > BI. Compared to conventional irrigation, AFI increased RWU from deeper and wetter root zones.

Root distribution is a crucial for plant uptake^18,44^, and as rooting depth increases water is taken from successive deeper soil depths^39,45^. Similar to Aina and Fapohunda (1986) for crops with fibrous roots, corn roots were mostly found in the 0–40 cm depth for BI. Thus, RWU of corn was mainly from the 0–40 cm soil depth during the growing season for BI^30,32^. In contrast, in AFI the maximum root density was in the depth of 40–80 cm at the late growing stage, which was in agreement with Li *et al*.^39^. A wider and deeper root distribution was obtained for AFI_(M/2)_ that also increased RWU from deeper soil zones.

Similar to this study, some previous studies also reported that plants prefer water uptake from near the soil surface after irrigation, but from deeper soil depths when the shallow soil water gets depleted^18,46–49^. Our results suggest that the amount of water that corn obtained from any soil layer and root zone depended on both soil water availability and root distribution. However, the leading factor affecting RWU of corn was the root distribution within the entire growing season and the soil moisture within a relatively shorter irrigation cycle.

### The reasons related to improving WUE by analyzing plant RWU pattern and soil water distribution

In this study, AFI increased corn yield when compared to BI. There was no significant yield reduction when only 50% of irrigation was applied (AFI_(M/2)_), and WUE substantially improved. The enhanced crop yields and WUE under deficit irrigation were possible because more water uptake took place from the wetter part of the corn root system that compensated for on the low uptake from the drier side.

This study showed that approximately 4–26% more water uptake was from the wetter side of AFI than CFI, suggesting that the RWU ability in AFI exceeded that in CFI. More water uptake was from the deeper soil depths of AFI than BI. AFI has greater potential for water savings by increasing the RWU from wetter as well as deeper soil depths. Our results are also in agreement with some previous studies^3,7,11^. Plants are able to modify their patterns of RWU in response to different levels of available water in their root zone.

Improved WUE for AFI and AFI_(M/2)_ could also be explained by analyzing the soil water profiles^2,3^. An upward gradient of soil water existed in the root zone of AFI. In contrast, a downward gradient existed in the root zone of CFI. Thus, water in AFI was stored in the root zone, decreasing deep percolation loss. No yield reductions in AFI_(M/2)_ during 2014 are difficult to explain, although it could be likely due to supply of water from deeper depths >110 cm^9^. However, yield reductions were noted in 2013, likely due to much lower precipitation of 68.4 mm in 2013 than 241.0 mm in 2014 (Table 1).

## Conclusion

This study used stable isotope analysis, soil water distribution, and root distribution to quantify corn water uptake and water use efficiency for four irrigation methods. A larger proportion of corn RWU from deeper soil layer was observed for AFI, and generally varied as AFI_(M/2)_ > AFI > CFI > BI. RWU pattern varied with the soil moisture within a short irrigation cycle and the root distribution over the growing season. AFI increased RWU by 4–26% from wetter root zones and decreased water loss to deep percolation, while increasing corn yield by 17.0–30.4%. Applying alternate furrow irrigation to field crops has greater potential for water savings, improving WUE, and maintaining yields. Therefore, the cause of enhanced crop yields and WUE under deficit irrigation can be attributed to its stimulation to root growth and promotion of a higher use efficiency of soil water with attendant decreases in water loss to deep percolation. Isotope analysis is shown to be an effective method to quantify RWU, and soil water distribution for different irrigation methods. AFI is an important technique for improving water use efficiency, and could play a crucial role in agricultural water management.
